# Arizona—New York

**Published:** 1902-10

**Authors:** 


### EDITORIALS.

### ARIZONA—NEW YORK.

It is refreshing, indeed, to read the modest account of what one active, earnest man can do in influencing for good public work in a State or Territory. When one considers what difficulties must necessarily confront veterinary sanitary control work in a Territory like Arizona, and learns of what has been accomplished in the control of infectious and contagious animal diseases, and to conjecture what immense sums of money have been saved to the people thereby, he is led to wonder how all of this great work could be accomplished by one active spirit; while, on the other hand, we are pained to read the confession of the President of the Association in the Empire State, acknowledging that their veterinary sanitary control work is still in the hands of laymen or ignorant pretenders, that their State Veterinary Practice law is worse than a dead letter; that their colleges are only half supported, while in numbers and personal success the profession is unusually numerous and very well provided with worldly goods and monetary resources.

